# The Perils of Overly Sensitive Viral Load Testing for Persons With Human Immunodeficiency Virus

**DOI:** 10.1093/ofid/ofad494

**Published:** 2023-10-03

**Authors:** Maria G Rodriguez, Alina Syros, Allan E Rodriguez, David P Serota

**Affiliations:** Division of Infectious Diseases, Department of Medicine, University of Miami Miller School of Medicine, Miami, Florida, USA; Division of Infectious Diseases, Department of Medicine, University of Miami Miller School of Medicine, Miami, Florida, USA; Division of Infectious Diseases, Department of Medicine, University of Miami Miller School of Medicine, Miami, Florida, USA; Division of Infectious Diseases, Department of Medicine, University of Miami Miller School of Medicine, Miami, Florida, USA

**Keywords:** HIV, treatment as prevention, virologic failure

## Abstract

The concept of “undetectable = untransmittable (U = U)” has been revolutionary in both the prevention and treatment of persons with human immunodeficiency virus (HIV). Most studies proving the concept of U = U used an HIV RNA (viral load [VL]) cutoff of 200 copies/mL to define being undetectable. Since then, increasingly sensitive commercial VL assays, sometimes down to a lower limit of detection (LLD) of 20 copies/mL, lead to confusion about the definition of “undetectable” and when someone is truly considered untransmittable. VLs between the LLD and 200 copies/mL have been associated with future virologic failure; however, no data exist to suggest that intervening in those patients leads to any meaningful benefits. In the absence of a demonstrable benefit of reporting such low VLs, we view this practice as harmful. We suggest recommendations for adjusting VL reporting and improving provider counseling, and call for research designs to mitigate the harms of overly sensitive VL testing.

## Teaser

Overly sensitive HIV viral load assays complicate the straightforward, transformative concept of “U=U”. In the absence of demonstrable benefits of reporting low viral load values (<200 copies/mL), we consider this to be a harmful practice and suggest mitigation strategies.

## CASE PRESENTATION

A young woman presented to a human immunodeficiency virus (HIV) primary care clinic for the treatment of newly diagnosed HIV infection. She was started on antiretroviral therapy (ART) and was counseled that with time and high adherence, she would achieve an “undetectable” HIV RNA level (viral load [VL]) and would be noninfectious to sexual partners. Over the subsequent 2 years, the patient's VL remained detectable, based on the laboratory's lower limit of detection (LLD) of 20 copies/mL, with measures between 20 and 200 copies/mL and a normal absolute CD4 lymphocyte count. She reported near-perfect adherence, which was confirmed by pharmacy refill records. Her physician explained that a sustained VL of <200 copies/mL meant that the virus was untransmittable; despite this explanation, she became increasingly frustrated about being unable to achieve an “undetectable” status based on reported laboratory values. She was skeptical about her lack of infectiousness, was dissatisfied with her ART, and was reticent to engage in sexual relationships, which she had avoided since her diagnosis.

## THE U = U MOVEMENT

“Undetectable = Untransmittable (U = U)” is the concept that persons with HIV (PWH) with an undetectable VL cannot transmit the virus to sexual partners [1, 2]. This concept of “treatment as prevention” (TasP) has been endorsed by health authorities across the globe. U = U developed from studies demonstrating a lack of HIV transmission in serodiscordant couples in which the individual with HIV was taking ART. The first study to assess TasP was the HIV Prevention Trials Network Study 052, demonstrating a significant decrease in HIV transmission in patients with early ART initiation [3]. Later observational studies focusing on both same-sex and opposite-sex partners have been essential to the establishment of 200 copies/mL as the lower limit of transmissibility [4]. A recent systematic review of the risk of sexual transmission from individuals with low-level viremia found no definitive evidence of HIV transmission when VLs were <600 copies/mL and an incredibly rare occurrence of possible transmissions with VLs between 600 and 1000 copies/mL [4].

Prior to the widespread consensus on U = U and the advent of HIV preexposure prophylaxis, HIV prevention in serodiscordant sexual partners relied upon counseling, frequent HIV testing, and emphasis on reduction of “risky behaviors” [5]. These older approaches to prevention were less effective and promoted the idea that sex with PWH was fundamentally more dangerous than with individuals without HIV. The development of TasP and U = U was novel in its understanding of HIV transmissibility, encouraging demand for increased HIV testing and early initiation of treatment [6]. Educating patients on U = U is associated with positive health outcomes, including improved perception of sexual and mental health and improved medication adherence [7–9]. Studies focusing on sexual minority men, a population disproportionately impacted by HIV, showed that PWH feel more comfortable about their HIV diagnosis when counseled in the context of U = U [10].

## OVERLY SENSITIVE VIRAL LOAD TESTING CHALLENGES

The definition of an *undetectable* VL has evolved over the years as clinical laboratory detection of HIV has advanced. Increasingly sensitive VL testing will inevitably lead to more PWH being labeled as “detectable,” when defined as “above the LLD of the assay” or even when the VL is below the LLD but still present and reported as detectable. Many commercial laboratories now detect as few as 20 viral copies/mL depending on which polymerase chain reaction (PCR) assay is used (Table 1). For patients, seeing a detectable VL reported but being told they are *untransmittable* sows confusion and may challenge beliefs in the otherwise clear U = U message, as it did in this patient case.

**Table 1.** Lower Limit of HIV Viral Load Detection and Reporting Guidelines of Select HIV-1 RNA Assays

HIV RNA Assay	VL Lower Limit of Detection, Copies/mL	Results Reported for Low VL Values
Roche COBAS AmpliPrep/COBAS TaqMan HIV-1 Test, version 2.0^a^	20	“HIV-1 RNA not detected” “HIV-1 RNA detected, <20 HIV-1 RNA copies/mL”
bioMérieux NucliSENS EasyQ HIV-1 v2.0^b^	10	“Target not detected” “<10 copies/mL”
Abbott RealTime HIV-1 Viral Load Assay^b^	40	“Not detected” “<40 copies/mL”
Hologic Aptima HIV-1 Quant Dx Assay^c^	30	“Not detected” “<30 detected”

Abbreviations: HIV, human immunodeficiency virus; VL, viral load.

^a^Roche Diagnostics. COBAS AmpliPrep/COBAS TaqMan HIV-1 Test, v2.0. https://diagnostics.roche.com/us/en/products/params/cobas-ampliprep-cobas-taqman-hiv-1-test-v2-0.html. Accessed 1 September 2023.

^b^Mourez T, Delaugerre C, Vray M, Lemee V, Simon F, Plantier JC. Comparison of the bioMérieux NucliSENS EasyQ HIV-1 v2.0-HIV-1 RNA quantification assay versus Abbott RealTime HIV-1 and Roche Cobas TaqMan HIV-1 v2.0 on current epidemic HIV-1 variants. *J Clin Virol* 2015; 71:76–81.

^c^Hologic. Aptima HIV-1 Quant Dx Assay. https://www.hologic.com/hologic-products/molecular-diagnostics/aptima-hiv-1-quant-dx-assay? utm_source=google&utm_medium=cpc&utm_term=hologic%20hiv&utm_campaign=2023_Brand_Virology&gclid=Cj0KCQjw6KunBhDxARIsAKFUGs-k9ozcNKiDHE3PP6kZTMr7GKRFgxGT__EB-3Za9gBeVzYOTLKzEs8aAlkLEALw_wcB. Accessed 1 September 2023.

Persistent HIV RNA level ≥200 copies/mL, and especially >500 copies/mL, has been associated with evidence of viral evolution and accumulation of drug resistance mutations [11, 12]. Patients who have a persistent HIV RNA level ≥200 copies/mL are considered to be experiencing virologic failure. The goal of ART is to suppress HIV replication as much as possible to a level below which drug resistance mutations cannot emerge. To date, there are no clear data indicating that a VL between the LLD and 200 copies/mL is clinically relevant in portending negative outcomes such as CD4 T-cell decline, cardiovascular disease, malignancy, or immunologic conditions associated with HIV infection. The main identified risk of this low-level viremia (LLV, variably defined as 20–200 copies/mL on ≥2 occasions) is an association with future virologic failure [13, 14]. It is also important to consider that VL assays lose precision at lower values of HIV RNA, potentially contributing to the observation of values that are continuously above the LLD [15, 16]. Yet it has not been established that identification of LLV or virological blips (single instances of detectable VLs <200 copies/mL) lead to actions that successfully prevent future virologic failure. Although LLV is associated with future virologic failure, in absolute terms, most virologic failures (>70%) actually occur among those without preceding LLV [13, 17].

Because VLs deemed detectable but <200 copies/mL have not demonstrated meaningful clinical implications, and have the potential to cause confusion and mistrust among patients and providers alike, we consider reporting these values to be a harmful medical practice with a negative public health message.

## NEXT STEPS FOR VIRAL LOAD REPORTING

**Figure 1.**
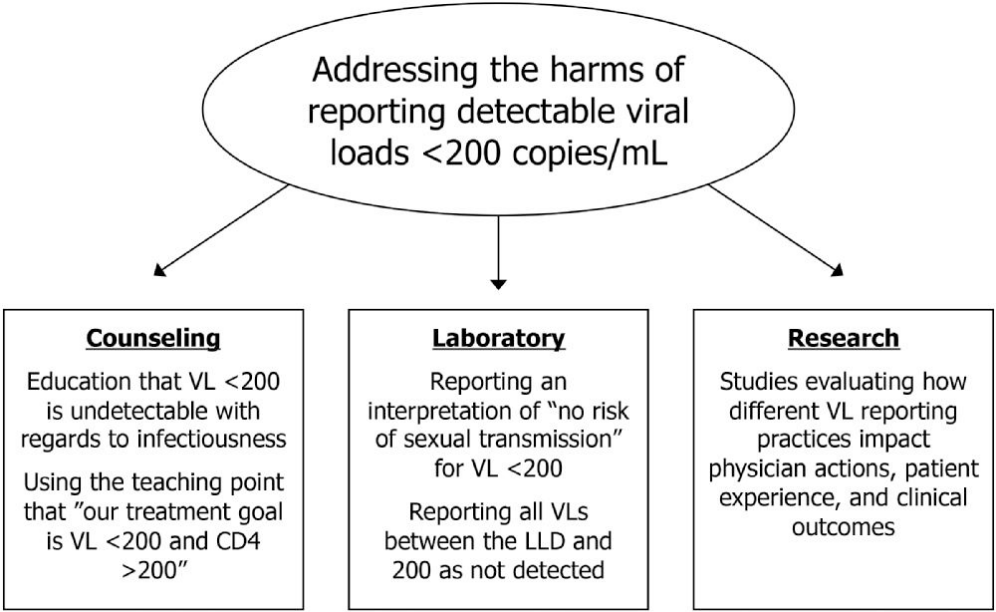
Approaches to overly sensitive viral load testing. Abbreviations: CD4, absolute CD4-positive lymphocyte count; LLD, lower limit of detection; VL, viral load.

The simplest approach to mitigating the harms of overly sensitive VL reporting lies in innovations in clinical laboratory reporting and electronic health record (EHR) display of results to patients and providers (Figure 1). One possibility is for laboratories and EHRs to continue reporting quantitative VLs with the addition of interpretation of the potential for sexual transmission. Physicians would know a patient's exact VL, but patients would have the benefit of a laboratory interpretation of “no risk of sexual transmission” if a VL is <200 copies/mL. Alternatively, all VLs <200 copies/mL could be automatically reported as *undetectable*, with the precise value hidden but available to providers to disclose with further explanation if needed. This change would also allow for consistency in reporting between different laboratory assays and between different institutions.

Providers should adjust patient education and counseling to preempt confusion about U = U and detectable VLs <200 copies/mL. One approach is to spend extra time ensuring that the term *undetectable* is equated specifically to a VL <200 copies/mL rather than looking for the words “not detected” or a “<” symbol in laboratory results. Another option is to focus on the number 200 as being an important number to remember for PWH to understand the status of their HIV. Patients can be counseled that “our goal for treatment is a VL less than 200 and CD4 count above 200.”

Finally, we call for further research into the value of patient and provider awareness of VL values between the LLD and 200 copies/mL. Studies facilitated through the EHR could randomize patients to having quantitative values between the LLD and 200 copies/mL displayed in their results compared to having all values <200 copies/mL hidden and reported only as “undetectable” or “<200.” The outcomes of interest would be to investigate if either approach is associated with a lower incidence of virologic failure and differences in quality of life or perceived health status. Additionally, studies evaluating adherence support programs might evaluate if the magnitude of benefit is higher for preventing virologic failure among those with LLV compared to those who have VLs less than the LLD.

## CONCLUSIONS

The establishment of U = U and the evidence showing near-zero risk of sexual transmission even with LLV has been transformative and empowering for PWH. It also provides an opportunity to destigmatize PWH and promote adherence to ART through dissemination of this positive public health message. Testing and reporting of VL values between the LLD and 200 copies/mL have the potential to sow confusion and further stigma in the absence of evidence that this information is clinically useful or actionable. We advocate for alternative approaches to reporting VL values, suggest adjusting patient counseling to clarify the definition of *undetectable*, and call for pragmatic research to determine the value—or harm—of highly precise VL reporting.

## Notes

***Author contributions*.** M. G. R. and A. S. were both third-year medical students at the time of drafting this manuscript. They conceptualized the article and performed writing of the original draft and writing–reviewing and editing of the final article. D. P. S. and A. E. R. helped conceptualize the article and edited the manuscript drafts.

***Patient consent*.** The case presented above is a fictitious case; therefore, no patient consent is required.

## References

[1] Eisinger RW , Dieffenbach CW, Fauci AS. HIV viral load and transmissibility of HIV infection: undetectable equals untransmittable. JAMA 2019; 321:451–2. 30629090 10.1001/jama.2018.21167

[2] Weld ED . Limits of detection and limits of infection: quantitative HIV measurement in the era of U = U. J Appl Lab Med 2021; 6:324–6. 33438739 10.1093/jalm/jfaa176 PMC8935681

[3] Cohen MS , Chen YQ, McCauley M, et al Antiretroviral therapy for the prevention of HIV-1 transmission. N Engl J Med 2016; 375:830–9. 27424812 10.1056/NEJMoa1600693 PMC5049503

[4] Broyles LN , Luo R, Boeras D, Vojnov L. The risk of sexual transmission of HIV in individuals with low-level HIV viraemia: a systematic review. Lancet 2023; 402:464–71. 37490935 10.1016/S0140-6736(23)00877-2 PMC10415671

[5] Muessig KE , Cohen MS. Advances in HIV prevention for serodiscordant couples. Curr HIV/AIDS Rep 2014; 11:434–46. 25145645 10.1007/s11904-014-0225-9 PMC4267973

[6] Smith P , Buttenheim A, Schmucker L, Bekker LG, Thirumurthy H, Davey DLJ. Undetectable = untransmittable (U = U) messaging increases uptake of HIV testing among men: results from a pilot cluster randomized trial. AIDS Behav 2021; 25:3128–36. 34057659 10.1007/s10461-021-03284-y PMC8165342

[7] Okoli C , Van de Velde N, Richman B, et al Undetectable equals untransmittable (U = U): awareness and associations with health outcomes among people living with HIV in 25 countries. Sex Transm Infect 2021; 97:18–26. 32732335 10.1136/sextrans-2020-054551 PMC7841488

[8] Thomford NE , Mhandire D, Dandara C, Kyei GB. Promoting undetectable equals untransmittable in sub-Saharan Africa: implication for clinical practice and ART adherence. Int J Environ Res Public Health 2020; 17:6163. 32854292 10.3390/ijerph17176163 PMC7503341

[9] Agaku I , Nkosi L, Gwar JN, Tsafa T. A cross-sectional analysis of U = U as a potential educative intervention to mitigate HIV stigma among youth living with HIV in South Africa. Pan Afr Med J 2022; 41:248. 35734331 10.11604/pamj.2022.41.248.33079 PMC9188005

[10] Rendina HJ , Talan AJ, Cienfuegos-Szalay J, Carter JA, Shalhav O. Treatment is more than prevention: perceived personal and social benefits of undetectable = untransmittable messaging among sexual minority men living with HIV. AIDS Patient Care STDS 2020; 34:444–51. 33064015 10.1089/apc.2020.0137 PMC7585600

[11] Aleman S , Söderbärg K, Visco-Comandini U, Sitbon G, Sönnerborg A. Drug resistance at low viraemia in HIV-1-infected patients with antiretroviral combination therapy. AIDS 2002; 16:1039–44. 11953470 10.1097/00002030-200205030-00010

[12] Karlsson AC , Younger SR, Martin JN, et al Immunologic and virologic evolution during periods of intermittent and persistent low-level viremia. AIDS 2004; 18:981–9. 15096800 10.1097/00002030-200404300-00005

[13] Fleming J , Mathews WC, Rutstein RM, et al Low-level viremia and virologic failure in persons with HIV infection treated with antiretroviral therapy. AIDS 2019; 33:2005–12. 31306175 10.1097/QAD.0000000000002306 PMC6774874

[14] Alvarez Estevez M , Chueca Porcuna N, Guillot Suay V, et al Quantification of viral loads lower than 50 copies per milliliter by use of the Cobas AmpliPrep/Cobas TaqMan HIV-1 test, version 2.0, can predict the likelihood of subsequent virological rebound to >50 copies per milliliter. J Clin Microbiol 2013; 51:1555–7. 23390288 10.1128/JCM.00100-13 PMC3647906

[15] Wiesmann F , Ehret R, Naeth G, et al Multicenter evaluation of two next-generation HIV-1 quantitation assays, Aptima Quant Dx and Cobas 6800, in comparison to the RealTime HIV-1 reference assay. J Clin Microbiol 2018; 56:e00292-18. 10.1128/JCM.00292-18 PMC6156314 30068537

[16] Park Y , Roh J, Kim S. Performance evaluation of the Aptima assays in comparison with the Cobas 6800 assays for the detection of HIV-1, HBV, and HCV in clinical samples. Ann Lab Med 2022; 42:447–56. 35177565 10.3343/alm.2022.42.4.447 PMC8859551

[17] Elvstam O , Malmborn K, Elen S, et al Virologic failure following low-level viremia and viral blips during antiretroviral therapy: results from a European multicenter cohort. Clin Infect Dis 2023; 76:25–31. 36100984 10.1093/cid/ciac762 PMC9825828

